# Early Evidence of Geographic Variation in Medicare Participation Among Newly Eligible Mental Health Providers Following the 2024 Coverage Expansion

**DOI:** 10.1111/jrh.70180

**Published:** 2026-07-03

**Authors:** Janessa M. Graves, Natalia V. Oster, Lisa A. Garberson, Davis G. Patterson, C. Holly A. Andrilla

**Affiliations:** ^1^ WWAMI Rural Health Research Center Department of Family Medicine School of Medicine University of Washington Seattle Washington USA

**Keywords:** access, Medicare, mental health, older adults, rural

## Abstract

**Purpose:**

To assess changes in marriage and family therapist (MFT) and mental health counselor (MHC) workforce distribution from April 2022 to October 2024, evaluating early impacts of Medicare's coverage expansion on provider participation in underserved rural communities.

**Methods:**

This repeat cross‐sectional study used Medicare Fee‐for‐Service Public Provider Enrollment Files and National Provider Identifier Registry data. Counties were categorized as metropolitan and rural (micropolitan or noncore) using Urban Influence Codes. We analyzed quarterly changes in Medicare participation and the proportion of counties with at least one participating provider across geographic categories.

**Results:**

From April 2022 to October 2024, MFTs participating in Medicare increased from 111 to 9394, and MHCs increased from 4013 to 24013. Initially, participation rates were low and did not differ significantly by rurality. By October 2024, participation rates were higher in rural versus metropolitan counties for both provider types (MFTs: 16.4% rural vs. 11.0% metropolitan; MHCs: 12.1% vs. 9.2%; both *p* < 0.001). The percentage of counties with at least one Medicare‐participating MFT increased from 2.5% to 26.5%, and from 27.2% to 55.8% for MHCs. However, only 7.4% of noncore counties had at least one Medicare‐participating MFT in October 2024, and 32.2% had at least one MHC.

**Conclusions:**

Medicare participation among MFTs and MHCs increased dramatically following the 2024 coverage expansion, especially among rural providers. However, absolute provider availability in rural counties remains low, underscoring the need for additional strategies to translate participation gains into meaningful improvements in rural mental health access.

## 1 Introduction

Rural America is facing a persistent mental health workforce crisis that threatens access to essential behavioral health services. As of March 2024, the Health Resources and Services Administration (HRSA) had designated 3862 Mental Health Professional Shortage Areas in rural areas, reflecting a workforce distribution that has left entire counties without adequate mental health resources. Residents of these counties must travel significantly farther than their urban counterparts to receive mental health services [1, 2, 3]. As a result, residents of rural areas are less likely to receive mental health services than their urban peers [4, 5]. For example, Medicare beneficiaries with a mental health condition who reside in rural areas had 1.13 fewer outpatient visits (in‐person or by telehealth) than urban‐residing beneficiaries in 2017 [6].

This access gap is particularly consequential for older adults who disproportionately rely on Medicare for their healthcare coverage. Access to mental health services through Medicare depends both on coverage and the availability of providers who participate in Medicare, and rural communities have historically had fewer Medicare‐participating mental health providers than urban areas [7]. This limited provider availability compounds other well‐documented barriers to care, such as rural older adults’ access to mental health services, including limited knowledge of existing resources and services and how to access them [8], stigma and personal beliefs regarding their need for mental health services [9], and psychosocial stressors, such as social isolation and loneliness [9, 10, 11].

Recognizing the urgent need to expand the behavioral health workforce, the Consolidated Appropriations Act of 2023 expanded eligibility to marriage and family therapists (MFTs) and mental health counselors (MHCs) to bill the Centers for Medicare and Medicaid Services (CMS). This policy change, effective January 1, 2024, represents the first addition of new provider types to Medicare's behavioral health coverage in decades [12], with over 300,000 MFTs and MHCs estimated now eligible to participate as Medicare providers. The timing of this expansion is particularly critical given that the COVID‐19 pandemic exacerbated existing workforce shortages by increasing demand for mental health care [13] while simultaneously accelerating provider burnout and attrition [14, 15]. Still, the population of adults over age 65 in rural areas continues to grow [16].

Previous research demonstrates that policies to increase the availability of mental health services are associated with increased utilization of these services [17]. Given the disproportionate impact of the Medicare mental health coverage gap in rural communities [18], it is reasonable to suspect that expansion of the Medicare mental health workforce to include MFTs and MHCs will increase provider participation and, ultimately, access to services in rural areas. Yet, the influence of Medicare's coverage expansion on MFT and MHC Medicare participation remains unknown. This study examines changes in Medicare participation of the MFT and MHC workforce from April 2022 to October 2024, providing a comprehensive evaluation of early impacts of Medicare's coverage expansion during its inaugural year.

Our analysis provides national‐level insights into provider participation in Medicare, with a specific emphasis on how changes over time may start to address longstanding incongruences in mental health care access across the rural‐urban spectrum.

## 2 Methods

Using a repeated cross‐sectional study design, we examined the quarterly supply of MFTs and MHCs in the U.S. from April 2022 to October 2024 using publicly available data from the Medicare Fee‐for‐Service Public Provider Enrollment Files (PPEF), the CMS National Plan and Provider Enumeration System (NPPES) National Provider Identifier (NPI) Registry, Medicare Monthly Enrollment data, and the 2013 Economic Research Service (ERS) Urban Influence Codes (UIC) [19]. This analysis was restricted to Medicare Fee‐for‐Service providers because the PPEF captures provider participation in traditional Medicare only and does not include MFTs and MHCs that contract directly with Medicare Advantage plans.

For each quarter, we identified MFTs and MHCs using the PPEF and taxonomy codes 106H00000X and 101YM0800X, respectively, and linked these data to the NPPES NPI registry to obtain provider ZIP Codes. We assigned each provider to a county using the U.S. Department of Housing and Urban Development ZIP Code Crosswalk Files and categorized counties using the 2013 UIC classification as metropolitan (UIC 1–2), micropolitan (UIC 3, 5, 8), or noncore (UIC 4, 6, 7, 9–12), with micropolitan and noncore counties considered rural.

For each quarter from April 2022 to October 2024, we calculated Medicare participation as the proportion of NPI‐registered MFTs and MHCs participating in Medicare, overall and within each geographic category. To assess geographic changes over time, we calculated the percentage of counties with at least one MFT or MHC participating in Medicare in April 2022 and October 2024, overall and by geographic category. We compared the percentage of providers participating in Medicare and the percentage of counties without any participating providers across geographic categories using chi‐square tests.

Because MFTs and MHCs were not eligible to participate in Medicare as independent providers prior to January 1, 2024, observed participation *before* this date reflects providers who held another Medicare‐eligible credential (e.g., clinical social worker) at that time. Therefore, while we refer to them as “participating in Medicare” because they were identifiable in the PPEF, these providers were not eligible to bill as MFTs or MHCs until January 2024. We used SAS System for Windows version 9.4 for data management and Stata/MP version 15.1 for analyses. The University of Washington Human Subjects Division determined this research to be exempt from human subjects review.

## 3 Results

**FIGURE 1:**
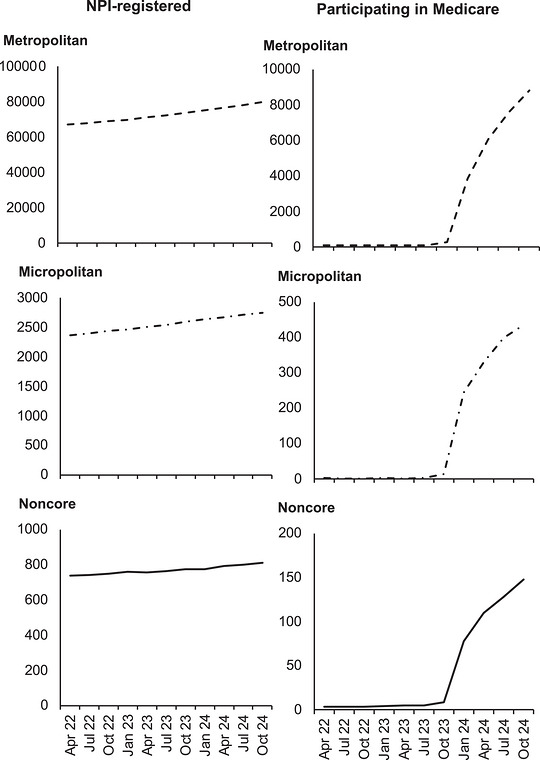
Number of NPI‐registered and Medicare‐participating MFTs, by geographic location, April 2022 to October 2024.

**FIGURE 2:**
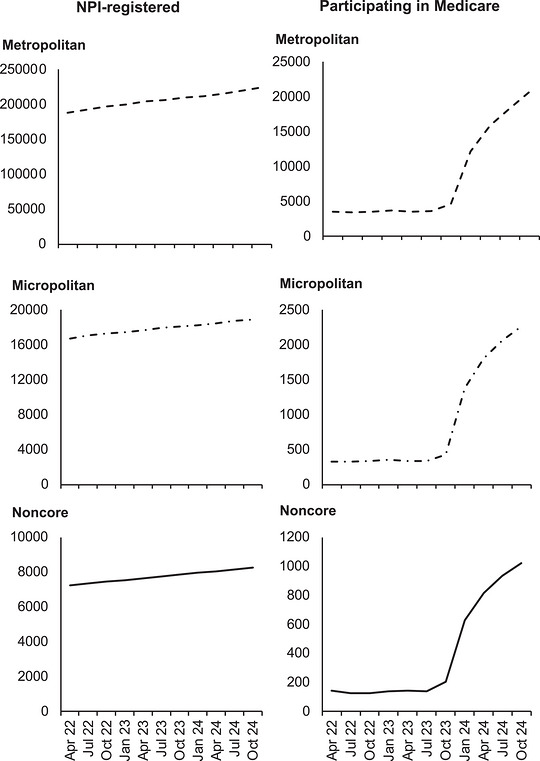
Number of NPI‐registered and Medicare‐participating MHCs, by geographic location, April 2022 to October 2024.

From April 2022 to October 2024, the number of NPI‐registered MFTs and MHCs in the U.S. increased gradually, while Medicare participation rose sharply following October 2023, when providers could begin the administrative process of enrolling in advance of the January 1, 2024 policy implementation date (Figures 1 and 2). Among MFTs, Medicare participation increased from 111 of 70,318 in April 2022 to 9394 of 83,464 in October 2024. Among MHCs, participation increased from 4013 of 212,056 in April 2022 to 24,013 of 251,615 in October 2024.

Across geographic categories, Medicare participation rates were essentially zero in April 2022 and not significantly different for either provider type (MFTs: *p* = 0.157; MHCs: *p* = 0.687) (Table 1). By January 2024, Medicare participation rates were significantly higher among providers in micropolitan and noncore counties than among those in metropolitan counties for both provider types (Table 1; MFTs: *p* < 0.001; MHCs: *p* < 0.001). By October 2024, Medicare participation rates among MFTs were 16.4% in rural counties (micropolitan and noncore together) and 11.0% in metropolitan counties. Among MHCs, Medicare participation rates were 12.1% in rural counties and 9.2% in metropolitan counties by October 2024.

**TABLE 1:** Medicare participation rates among NPI‐registered MFTs and MHCs, by geographic location, April 2022 to October 2024.

	Marriage and Family Therapists					Mental Health Counselors				
			Rural Counties					Rural Counties		
	Metro Counties	Rural Counties	Micro	Noncore	Total	Metro Counties	Rural Counties	Micro	Noncore	Total
Apr 22	0.2%	0.2%	0.1%	0.4%	0.2%	1.9%	2.0%	2.0%	1.9%	1.9%
Jul 22	0.1%	0.1%	0.0%	0.4%	0.1%	1.8%	1.9%	1.9%	1.7%	1.8%
Oct 22	0.1%	0.1%	0.0%	0.4%	0.1%	1.8%	1.9%	1.9%	1.7%	1.8%
Jan 23	0.1%	0.2%	0.1%	0.5%	0.2%	1.9%	2.0%	2.0%	1.8%	1.9%
Apr 23^a^	0.1%	0.2%	0.0%	0.7%	0.1%	1.7%	1.9%	1.9%	1.8%	1.8%
Jul 23^a^	0.1%	0.2%	0.1%	0.7%	0.1%	1.7%	1.8%	1.9%	1.8%	1.7%
Oct 23^a^	0.4%	0.7%	0.5%	1.0%	0.4%	2.2%	2.4%	2.4%	2.6%	2.2%
Jan 24^a^	5.1%	9.4%	9.2%	10.1%	5.3%	5.7%	7.7%	7.6%	7.9%	5.9%
Apr 24^a^, ^b^	7.9%	12.7%	12.3%	13.9%	8.1%	7.4%	9.9%	9.8%	10.1%	7.6%
Jul 24^a^, ^b^	9.7%	15.0%	14.7%	16.0%	9.9%	8.4%	11.1%	11.0%	11.5%	8.7%
Oct 24^a^, ^b^	11.0%	16.4%	15.8%	18.3%	11.3%	9.2%	12.1%	11.9%	12.4%	9.5%

*Abbreviations*: Metro, metropolitan; Micro, micropolitan. Geographic categories based on 2013 UIC; with metropolitan counties are considered urban, while micropolitan and noncore counties are considered rural.

^a^ Chi‐square test of independence across metropolitan, micropolitan, and noncore counties, MFT: p<.001

^b^ Chi‐square test of independence across metropolitan, micropolitan, and noncore counties, MHC: p<.001

**FIGURE 3:**
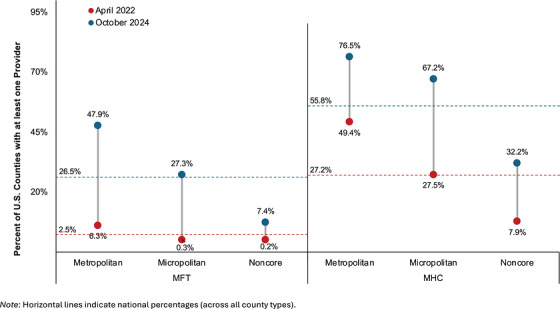
Percentage of U.S. counties with at least one MFT (left panel) or MHC (right panel) participating in Medicare in April 2022 and October 2024, across geographic rurality categories.

In April 2022, 2.5% of U.S. counties had at least one Medicare‐participating MFT, which increased to 26.5% in October 2024, representing a gain of 24.0 percentage points (Figure 3). The percentage of U.S. counties with at least one Medicare‐participating MHC increased from 27.2% to 55.8% between April 2022 and October 2024, representing a doubling of the number of counties with at least one Medicare‐participating MHC. The drastic increase in the percentage of counties with at least one MFT or MHC participating in Medicare between April 2022 and October 2024 varied by rurality. The change in the percentage of micropolitan and noncore counties with at least one Medicare‐participating MFT (27.0 and 7.2 percentage points, respectively) was smaller than in metropolitan counties (41.6). The gains in the percentage of counties with at least one Medicare‐participating MHC were greatest in micropolitan counties (39.7 percentage points), followed by metropolitan counties (27.1) and noncore counties (24.3).

## 4 Discussion

This study identified a dramatic increase in Medicare participation among MFTs and MHCs after Medicare expanded coverage to these provider types in January 2024. Between April 2022 and October 2024, combined MFT and MHC participation surged from 4142 to 33,407 providers, validating the relevance of this policy intervention. Notably, participation gains accelerated after October 2023, when providers could start enrolling under the new reimbursement rules, suggesting that many were ready to take immediate action upon policy implementation. This rapid uptake by “early adopters” is encouraging, yet it also highlights the ongoing need for outreach and assistance to providers who may require additional support, such as independent and small‐group practitioners who may lack the billing infrastructure to navigate Medicare participation without assistance. This need has been recognized by groups like the Medicare Mental Health Workforce Coalition, the National Board for Certified Counselors, and the American Association for Marriage and Family Therapy through their educational webinar initiatives and materials [20, 21, 22].

A particularly notable finding from this study is the geographic distribution of Medicare participation rates following the January 2024 policy implementation. Prior to the policy change, participation rates were uniformly low and statistically indistinguishable across metropolitan, micropolitan, and noncore counties for both provider types. This low participation rate is to be expected, given that MFTs and MHCs were not eligible to bill Medicare, and those identified in Medicare PPEF data represent providers with another license or credential that was eligible to bill Medicare. By October 2024, however, Medicare participation rates among both MFTs and MHCs were significantly higher in rural counties than in metropolitan counties, with noncore MFTs showing the highest participation rate compared to their metropolitan counterparts. The outpacing of participation by rural versus metropolitan providers was a consistent pattern across both provider types throughout all post‐implementation quarters. One plausible explanation for this pattern is that Medicare may make up a proportionally larger share of rural MFTs’ and MHCs’ potential reimbursement base (given the higher percentage of Medicare beneficiaries in rural vs. urban areas) [23], and thus they may have a stronger financial incentive to participate. Further, rural providers may be more likely than urban providers to operate in small practices [24, 25] and thus have greater flexibility to adapt their billing arrangements in a timely manner.

Beyond its implications for access, this policy change carries significant meaning for MFTs and MHCs as a profession. Prior to January 2024, these providers were ineligible to bill Medicare, limiting their ability to serve Medicare beneficiaries, which is especially relevant in rural communities where Medicare represents a large share of the insured population. Eligiblity to participate in Medicare represents formal federal recognition of MFTs and MHCs as independent behavioral health providers and opens the door for these practicing mental health providers to serve their communities’ needs [26, 27]. At the same time, this expansion raises an important concern regarding workforce readiness: MFT and MHC preparation programs have historically provided limited training on counseling older adults [28, 29]. Intentional integration of gerontological content into training curricula will be essential to ensuring that newly eligible providers are equipped to meet the complex behavioral health needs of rural Medicare beneficiaries [28].

The percentage gains in Medicare participation reported in this study must be interpreted carefully, given the low baseline numbers in micropolitan and non‐core rural counties, where many areas started with relatively few providers (Table S1). When participation begins near zero, even a small absolute increase generates large percentage gains. Therefore, while improvements in rural counties may appear substantial, they still represent only a small number of providers serving areas where they are sorely needed. This is reflected in the October 2024 snapshot: only 7.4% of noncore counties had at least one MFT participating in Medicare, and just 32.2% had at least one MHC, even though provider‐level Medicare participation rates in noncore counties were 18.3% among MFTs and 12.4% among MHCs. Nonetheless, the accelerated participation in Medicare among rural providers represents an encouraging early signal that this workforce expansion policy could begin to address inequities in behavioral health provider availability across the rural‐urban continuum. Beyond geographic disparities in participation rates, questions remain about whether gains in participation may be concentrated in areas already served rather than meaningfully expanding access to underserved or sparsely populated regions.

Several important considerations emerge when interpreting these findings. Our analysis captures only the inaugural year of policy implementation and may not reflect long‐term workforce distribution patterns or provider practice decisions. Also, this study examines only MFT and MHC participation in Medicare, which predominantly covers adults 65 and older and individuals with disabilities, and therefore, our findings speak specifically to behavioral health access for older adults and their families. Future research should explore the impact of the Medicare coverage expansion on participation in Medicaid and other payer contexts, where access to behavioral health services is equally critical for younger families and children. Although providers presumably participate with the intention of providing services to Medicare beneficiaries, it remains unclear the extent to which MFTs’ and MHCs’ participation in Medicare will translate into actual provision of care. Longitudinal studies examining potential changes in patient utilization patterns could help monitor downstream impact of this policy.

Our national perspective may mask important differences in Medicare participation within counties, particularly in geographically large rural counties where there are few providers and/or providers tend to cluster in more populated areas. Additionally, our analysis relies on addresses reported by providers to the NPPES, which may not reflect their actual practice locations or practice patterns across multiple locations, potentially misrepresenting the true geographic distribution of services. This analysis also focuses on participation in Medicare Fee‐for‐Service plans and does not capture the full picture of potential access improvements, as it does not capture MFTs and MHCs who may work with Medicare Advantage plans also or instead. This exclusion is particularly notable given the known geographic variation in Medicare Advantage penetration [30]. Together, these limitations suggest that the present analysis may underestimate the full scope of MFT and MHC participation in the Medicare ecosystem.

## 5 Conclusions

Several considerations for maximizing the impact of Medicare's mental health coverage expansion emerge from our findings. The persistent differences observed in workforce maldistribution across geography suggest that complementary policy approaches beyond simply expanding eligible provider types may be necessary to ensure equitable access to mental health services. Such approaches might include loan forgiveness or repayment programs, expanded rural training opportunities, and other location‐based incentives to encourage providers to practice in rural areas. Expanding the pipeline of MFTs and MHCs trained in older adult care will be essential to translate early gains in Medicare participation into meaningful improvements in access.

The expansion of Medicare coverage to include MFTs and MHCs may represent progress toward addressing critical mental health workforce shortages, particularly important given the growing rural older adult population and well‐documented gaps in access to mental health services. While geographic differences persist and challenges remain in ensuring access across urban and rural communities, the historic expansion of Medicare's behavioral health workforce provides an important step forward in strengthening mental health care access for Medicare beneficiaries nationwide.

## Funding

This study was supported by the Federal Office of Rural Health Policy (FORHP), the Health Resources and Services Administration (HRSA), and the U.S. Department of Health and Human Services (HHS) under cooperative agreement #U1CRH03712. The information, conclusions, and opinions expressed in this paper are those of the authors, and no endorsement by FORHP, HRSA, or HHS is intended or should be inferred.

## Disclosures

Authors have no disclosures to declare.

## Conflicts of Interest

The authors declare no conflicts of interest.

## Supporting information




**Supplemental Table 1**: Number of NPI‐registered and Medicare‐participating MFTs and MHCs, by geographic category, April 2022 to October 2024.
