# Supplementary material for: Early Evidence of Geographic Variation in Medicare Participation Among Newly Eligible Mental Health Providers Following the 2024 Coverage Expansion
Source: J Rural Health. 2026 Jul 3;42(3):e70180. doi: 10.1111/jrh.70180 (PMC13329745; doi:10.1111/jrh.70180)
Supplement: Supplementary file 1 — Supplemental Table 1: Number of NPI‐registered and Medicare‐participating MFTs and MHCs, by geographic category, April 2022 to October 2024. [file JRH-42-0-s001.docx]

**Supplemental Table 1.** Number of NPI-registered and Medicare-participating MFTs and MHCs, by geographic category, April 2022 to October 2024.

|  | **MFTs with registered NPI** | | | | | **MFTs participating in Medicare** | | | | | | |
| --- | --- | --- | --- | --- | --- | --- | --- | --- | --- | --- | --- | --- |
|  | **Metro Counties** | **Rural Counties** | **Rural Counties** | | **Total** | **Metro Counties** | **Rural Counties** | **Rural Counties** | | **Total** | | |
|  |  |  | **Micro** | **Noncore** |  |  |  | **Micro** | **Noncore** |  | |  |
| Apr 22 | 67,217 | 3,101 | 2,364 | 737 | 70,318 | 106 | 5 | 2 | 3 | 111 | |  |
| Jul 22 | 68,057 | 3,142 | 2,400 | 742 | 71,199 | 99 | 4 | 1 | 3 | 103 | |  |
| Oct 22 | 69,081 | 3,185 | 2,438 | 747 | 72,266 | 99 | 4 | 1 | 3 | 103 | |  |
| Jan 23 | 69,870 | 3,225 | 2,467 | 758 | 73,095 | 104 | 6 | 2 | 4 | 110 | |  |
| Apr 23 | 71,088 | 3,263 | 2,506 | 757 | 74,351 | 100 | 6 | 1 | 5 | 106 | |  |
| Jul 23 | 72,401 | 3,305 | 2,541 | 764 | 75,706 | 99 | 7 | 2 | 5 | 106 | |  |
| Oct 23 | 73,782 | 3,372 | 2,597 | 775 | 77,154 | 284 | 22 | 14 | 8 | 306 | |  |
| Jan 24 | 75,100 | 3,416 | 2,642 | 774 | 78,516 | 3,863 | 322 | 244 | 78 | 4,185 | |  |
| Apr 24 | 76,691 | 3,465 | 2,673 | 792 | 80,156 | 6,055 | 439 | 329 | 110 | 6,494 | |  |
| Jul 24 | 78,049 | 3,519 | 2,719 | 800 | 81,568 | 7,584 | 527 | 399 | 128 | 8,111 | |  |
| Oct 24 | 79,906 | 3,558 | 2,749 | 809 | 83,464 | 8,812 | 582 | 434 | 148 | 9,394 | |  |
|  | **MHCs with registered NPI** | | | | | **MCHs participating in Medicare** | | | | | | |
|  | **Metro Counties** | **Rural Counties** | **Rural Counties** | | **Total** | **Metro Counties** | **Rural Counties** | **Rural Counties** | | **Total** | | |
|  |  |  | **Micro Counties** | **Noncore** |  |  |  | **Micro** | **Noncore** |  | |  |
| Apr 22 | 188,045 | 24,011 | 16,759 | 7,252 | 212,056 | 3,542 | 471 | 331 | 140 | 4,013 | |  |
| Jul 22 | 192,309 | 24,404 | 17,059 | 7,345 | 216,713 | 3,411 | 452 | 328 | 124 | 3,863 | |  |
| Oct 22 | 197,077 | 24,772 | 17,303 | 7,469 | 221,849 | 3,484 | 461 | 336 | 125 | 3,945 | |  |
| Jan 23 | 200,409 | 25,019 | 17,487 | 7,532 | 225,428 | 3,716 | 489 | 352 | 137 | 4,205 | |  |
| Apr 23 | 204,161 | 25,332 | 17,690 | 7,642 | 229,493 | 3,556 | 475 | 334 | 141 | 4,031 | |  |
| Jul 23 | 206,164 | 25,656 | 17,925 | 7,731 | 231,820 | 3,571 | 474 | 337 | 137 | 4,045 | |  |
| Oct 23 | 209,778 | 25,998 | 18,124 | 7,874 | 235,776 | 4,624 | 627 | 426 | 201 | 5,251 | |  |
| Jan 24 | 212,132 | 26,245 | 18,282 | 7,963 | 238,377 | 12,153 | 2,010 | 1,382 | 628 | 14,163 | |  |
| Apr 24 | 215,508 | 26,511 | 18,472 | 8,039 | 242,019 | 15,842 | 2,620 | 1,805 | 815 | 18,462 | |  |
| Jul 24 | 219,516 | 26,861 | 18,727 | 8,134 | 246,377 | 18,361 | 2,994 | 2,059 | 935 | 21,355 | |  |
| Oct 24 | 224,427 | 27,188 | 18,940 | 8,248 | 251,615 | 20,730 | 3,283 | 2,260 | 1023 | 24,013 | |  |
| *Abbreviations*: MFT, marriage and family therapist; MHC, mental health counselor. Metro, metropolitan; Micro, micropolitan. Geographic categories based on 2013 UIC; with metropolitan counties are considered urban, while micropolitan and noncore counties are considered rural. | | | | | | | | | | |  |  |
